# Prognostic Value of Plasma Pentraxin-3 Levels in Patients with Stable Coronary Artery Disease after Drug-Eluting Stent Implantation

**DOI:** 10.1155/2014/963096

**Published:** 2014-11-24

**Authors:** Liu Haibo, Guo Xiaofang, Wang Chunming, Yuan Jie, Chen Guozhong, Zhang Limei, Cao Yong, Fang Yu, Bao Yingchun, Yu Wangjun, Ge Junbo

**Affiliations:** ^1^Shanghai Institute of Cardiovascular Diseases, Zhongshan Hospital, Fudan University, Shanghai 200032, China; ^2^Department of Cardiology, Yinzhou People's Hospital Affiliated to Medical School of Ningbo University, Ningbo 315040, China; ^3^Clinical Laboratory Center, Yinzhou People's Hospital Affiliated to Medical School of Ningbo University, Ningbo 315040, China

## Abstract

Pentraxin-3 (PTX3) is an inflammatory marker thought to be more specific to cardiovascular inflammation than C-reactive protein (CRP). Our aim was to assess the prognostic value of PTX3 in patients with stable coronary artery disease (CAD) after drug eluting stent (DES) implantation. Plasma PTX3 levels were measured before percutaneous coronary intervention (PCI) and at 24 h post-PCI in 596 consecutive patients with stable CAD. Patients were followed up for a median of 3 years (range 1–5) for major adverse cardiovascular events (MACEs). We found that the post-PCI plasma PTX3 levels were significantly higher at 24 h after PCI than pre-PCI, patients with MACEs had higher post-PCI PTX3 levels compared with MACEs-free patients, patients with higher post-PCI PTX3 levels (median > 4.384 ng/mL) had a higher risk for MACEs than those with PTX3 < 4.384 ng/mL, and post-PCI PTX3, cTnI, multiple stents, and age but not high-sensitivity CRP (hsCRP) were independently associated with the prevalence of MACEs after DES implantation. The present study shows that post-PCI PTX3 may be a more reliable inflammatory predictor of long-term MACEs in patients with stable CAD undergoing DES implantation than CRP. Measurement of post-PCI PTX3 levels could provide a rationale for risk stratification of patients with stable CAD after DES implantation.

## 1. Introduction

Pentraxin-3 (PTX3) and C-reactive protein (CRP) are members of pentraxin family, and unlike CRP which is a short pentraxin synthesized in the liver in response to various inflammatory signals and cytokines and may represent a systemic response to local inflammation [1, 2], PTX3 is a long pentraxin produced mainly by dendritic cells, macrophages, and endothelial cells response directly to the local site of inflammation [3–6], and highly expressed in the cardiovascular system [7–9], and compared with CRP, PTX3 may be a more useful inflammatory marker for localized vascular inflammation and damage in the cardiovascular system.

Inflammation has been suggested to be associated with the adverse cardiovascular events in patients with coronary artery disease (CAD) [10–12]. Percutaneous coronary intervention (PCI) induces a significant inflammatory reaction in the injured vessel wall that leads to the adverse cardiovascular events [13, 14]; PCI with coronary stent implantation has become the most common treatment modality of CAD and there are two types of coronary stent: bare-metal stent (BMS) and drug-eluting stent (DES). Recent studies have shown that PTX3 levels were increased after coronary stenting [15–17] and PTX3 is associated with the cardiovascular events in patients after BMS implantation [17, 18]. However, DES can greatly reduce incidence of cardiovascular events because of its anti-inflammatory properties compared to BMS [19], whether PTX3 is still associated with the cardiovascular events after only DES implantation is not known. Hence, in this study, we aimed to assess the long-term prognostic value of PTX3 in patients with stable CAD after DES implantation.

## 2. Materials and Methods

### 2.1. Patient Population

Study patients were consecutively enrolled and undergoing PCI at Yinzhou People's Hospital, Medical school of Ningbo University, between July 2009 and June 2013. Patients were enrolled if they had implanted with DES for stable CAD. This study was approved by the ethics committees of Yinzhou People's Hospital, Medical school of Ningbo University, and all patients gave their written informed consent. Exclusion criteria included patients with acute coronary syndromes, renal dysfunction (serum creatinine > 1.5 mg/dL), severe hepatic or lung disease, chronic or acute inflammation, and malignant disease.

In this study, demographics and clinical data including age, gender, diabetes mellitus, hypertension, hyperlipidemia, current smoking, the number and type of coronary stents, Canadian Cardiovascular Society Angina (CCSA) class, and percentage of left ventricular ejection fraction (LVEF%) on admission were collected from in-hospital medical records and patient interviews.

### 2.2. Venous Blood Samples and Laboratory Analyses

Venous blood samples were collected before and at 24 h after PCI in all patients. Whole blood was immediately collected into a tube containing ethylene diaminetetraacetate (EDTA); then, it was centrifuged at 2000 ×g for 15 min at room temperature and the plasma was frozen for PTX3 and stored at −80°C until analysis. Plasma PTX3 concentrations were measured by enzyme-linked immunosorbent assay (ELISA; Perseus Proteomics Inc., Tokyo, Japan) as reported previously [20]. This assay can measure plasma PTX3 concentration linearly between 0.1 and 20 ng/mL. Plasma PTX3 levels were measured before and at 24 h after PCI, plasma hsCRP and cardiac troponin I concentrations were determined at 24 h after PCI.

### 2.3. Study Endpoint and Followup

All patients underwent clinical followup with a median period of 3 years (range 1–5) for MACEs after admission, the MACEs defined as (1) cardiac death, (2) nonfatal myocardial infarction (MI), and (3) target vessel revascularization (TVR). Clinical followup after PCI was performed via office visit or telephone contact at 1, 6, and 12 months and then every 6 months thereafter. All deaths were considered to be from cardiac causes unless an unequivocal noncardiac cause could be established. At these visits, data pertaining to patients' clinical status, all interventions, and adverse events were recorded. Clinical, procedural, and outcome data were prospectively collected by two authors (Zhang Limei and Cao Yong) who were blinded to PTX3 levels.

### 2.4. Statistical Analysis

All data analyses were performed using SPSS, version 17.0 (SPSS, Chicago, IL, USA). Continuous data are presented as mean ± SD and were compared with an unpaired Student's *t*-test, linear regression analysis, or Mann-Whitney *U*-test. Categorical variables are reported as frequencies (%) and were compared with the chi-square statistic or Fisher exact test, as appropriate. Relative risk with 95% confidence intervals (CI) is presented. Univariate and multivariate Cox proportional hazards regression were used to evaluate the prognostic value of clinical and biochemical variables. Survival analysis for patients between lower and higher PTX3 levels groups was performed using the Kaplan-Meier method, and the differences between groups were assessed by the log-rank test. A value of *P* < 0.05 was considered significant.

## 3. Results

### 3.1. Clinical Characteristics, Followup, and Plasma PTX3 Levels

The study group consisted of 596 patients (467 males, 129 females, mean age: 65.9 ± 8.1 years). DES implantation was performed successfully in all patients; types of DES used were sirolimus-eluting stents (65.5% of the population), zotarolimus-eluting stents (21.9%), paclitaxel-eluting stents (10.5%), and other DES (2.1%).

During the median of 3 years (range 1–5) of followup, the MACEs occurred in 82 (13.8%) patients, including 22 (3.7%) cardiac deaths, 14 (2.3%) nonfatal MI and 46 (7.7%) TVR.

**Figure 1:**
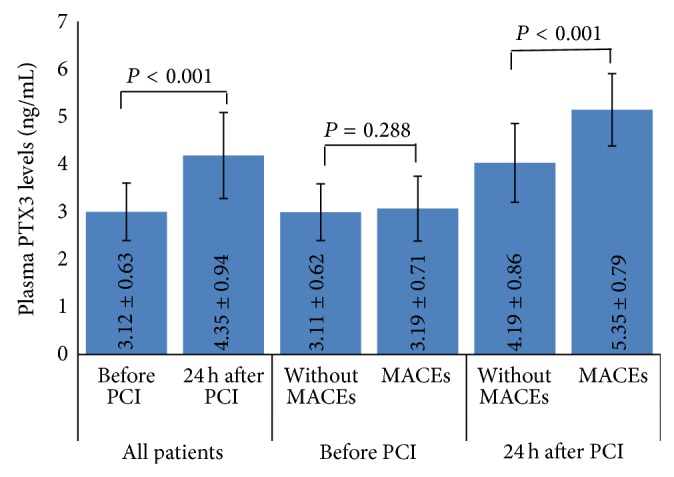
Plasma PTX3 levels in patients: 24 h after PCI versus before PCI in all patients, MACEs group versus without MACEs group before PCI, and MACEs group versus without MACEs group at 24 h after PCI.

Firstly, we wanted to know whether PTX3 levels were increased after DES implantation, then to assess the association between the PTX3 levels and MACEs after PCI. As can be seen from Figure 1, the plasma post-PCI PTX3 levels were significantly elevated at 24 h than pre-PCI (4.35 ± 0.94 versus 3.12 ± 0.63, *P* < 0.001) and pre-PCI PTX3 levels were not significant different between MACEs and events-free groups (3.19 ± 0.71 versus 3.11 ± 0.62, *P* = 0.288); however, 82 patients with MACEs had higher post-PCI PTX3 levels at 24 h after PCI compared with 514 events-free patients (5.36 ± 0.79 versus 4.19 ± 0.86, *P* < 0.001).

Table 1 showed patients with a cardiac event had higher concentrations of post-PCI hsCRP (*P* < 0.001) and cTnI (*P* < 0.001) compared with those without a cardiac event. Patients with a cardiac event had a higher prevalence of diabetes mellitus (*P* = 0.047), CCSA class > II (*P* = 0.015), LVEF < 50% (*P* = 0.037), multiple stents (*P* < 0.001), and higher age (*P* < 0.001).

### 3.2. Clinical Characteristics according to Median Value of PTX3

**Figure 2:**
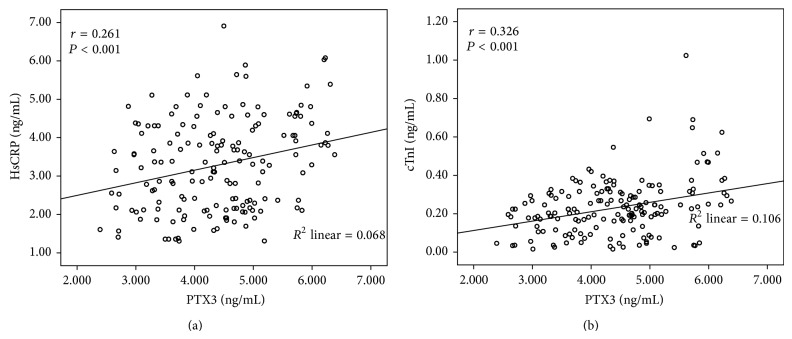
The relationship between PTX3 and hsCRP (a) and cTnI (b) by correlation analysis.

Patients were grouped into 2 according to media value of PTX3 at 24 h after PCI (4.384 ng/mL); the baseline clinical characteristics of the study groups are summarized in Table 2. Patients with elevated PTX3 (>4.384 ng/mL) had a higher prevalence of LVEF < 50% (*P* = 0.020), multiple stents (*P* = 0.024), and higher concentrations of hsCRP (*P* < 0.001) and CTnI (*P* < 0.001) compared to patients without elevated PTX3; there were no significant difference in other clinical characteristics between elevated PTX3 group and nonelevated PTX3 group. Correlation analysis revealed that the serum of PTX3 was weakly but significantly correlated with hsCRP (*R* = 0.261, *P* < 0.001) and cTnI (*R* = 0.326, *P* < 0.001) (Figure 2).

Table 3 displayed patients with elevated post-PCI PTX3 levels (>4.384 ng/mL) had a significant higher risk of nonfatal MI (*P* = 0.033), TVR (*P* < 0.001) and MACEs (*P* < 0.001) compared to nonelevated PTX3 levels group (<4.384 ng/mL); the prevalence of cardiac death was higher in elevated PTX3 levels compared with nonelevated PTX levels group (4.7% versus 2.7%), but there was no significant difference between them (*P* = 0.20).

### 3.3. Univariate and Multivariate Predictors of MACEs

PTX3 (*P* < 0.001), cTnI (*P* = 0.002), hsCRP (*P* < 0.001), age (*P* < 0.001), CCSA class > II (*P* = 0.011), LVEF < 50% (*P* = 0.024), Diabetes mellitus (*P* = 0.031) and multiple stents (*P* < 0.001) were significantly associated with MACEs by the univariate Cox regression analysis. In a multivariate Cox regression analysis including PTX3, cTnI, hsCRP, age, CCSA class > II, prevalence of LVEF < 50%, diabetes mellitus, and multiple stents, PTX3 (RR 2.512, *P* = 0.001; 95% CI, 1.466–4.305), cTnI (RR 1.012, *P* = 0.008; 95% CI, 1.004–1.284), multiple stents (RR 2.401, *P* < 0.001; 95% CI, 1.479–3.899), and age (RR 1.041, *P* = 0.013; 95% CI, 1.008–1.074) but not hsCRP (RR 1.093, *P* = 0.056; 95% CI, 0.998–1.197) were independently associated with the prevalence of MACEs (Table 4).

**Figure 3:**
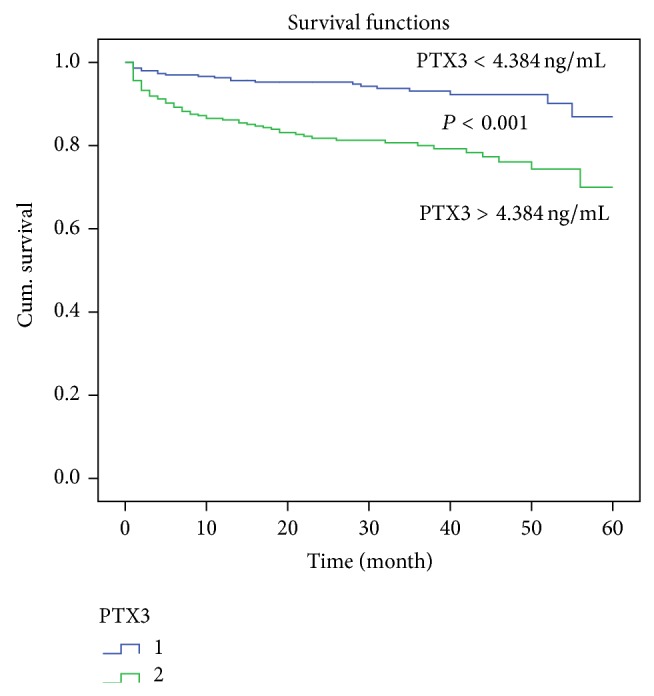
Kaplan-Meier curves showing incidence of cardiac event after PCI according to PTX3 (above versus below the median of 4.384 ng/mL) in patients undergoing coronary stenting. *P* value is calculated by log-rank test.

In the Kaplan-Meier analysis, patients with elevated PTX3 levels had a higher risk for MACEs than those patients without elevated PTX3 (*P* < 0.001) (Figure 3).

## 4. Discussion

In this cohort of patients with stable CAD after DES implantation and during the median of 3 years (range 1–5) followup, we found that (1) the plasma PTX3 levels were significantly increased at 24 h after PCI than before, (2) patients with elevated post-PCI PTX3 levels (above the median value of 4.384 ng/mL) had a significantly higher risk of MACEs, (3) patients with a cardiac event had higher concentrations of post-PCI PTX3, (4) PTX3, cTnI, hsCRP, age, CCSA class > II, LVEF < 50%, diabetes mellitus and multiple stents were significantly associated with cardiovascular events by univariate Cox regression analysis, and (5) in multivariate Cox regression analysis, the plasma concentration of post-PCI PTX3, but not hsCRP, remained an independent inflammatory predictor of cardiovascular events.

These findings suggest that post-PCI PTX3 may be a more reliable and independent inflammatory predictor for early risk stratification compared to hsCRP in patients with stable CAD after DES implantation, and measuring PTX3 may substantially improve the early risk stratification of patients after DES implantation. Such findings may have important implications for immediate management of high risk patients after DES implantation.

To our knowledge, we are the first to report an independent association of PTX3 with the MACEs among patients with stable CAD after DES implantation independence of systemic inflammation.

CRP is a widely used marker of inflammation and was associated with clinical worse outcomes in patients after PCI [21]. PTX3 and CRP are members of pentraxin family; CRP is a short pentraxin produced in the liver in response to various inflammatory signals and cytokines and may represent a systemic response to local inflammation [1, 2]; however, PTX3 is a long pentraxin produced mainly by dendritic cells, macrophages, endothelial cells, smooth muscle cells, and fibroblasts at the site of inflammation [3–6]. PCI induced inflammatory reaction in the injured vessel wall that could lead to the increase of both PTX3 and CRP, but based on the different mechanisms of induction of PTX3 and CRP, PTX3 should more directly reflect the local inflammatory response in the injured vessel wall during PCI.

In the present study, we found that PTX3 levels were significantly elevated at 24 h after PCI than before and there were several studies have shown a rise in PTX3 during PCI. Kotooka et al. [17] reported that the plasma PTX3 levels began to increase at 15 min after coronary stenting and reached a maximum at 24 h in the coronary sinus and peripheral blood. Other 2 studies also found PCI could mediate the increase of PTX3 [15, 16].

PCI induced inflammatory reaction in the injured vessel wall that leads to the adverse cardiovascular events [13, 14]. There are some researches indicated that inflammatory markers, such as PTX3 and CRP, elevated in patients after PCI and these inflammatory proteins predicted the clinical worse outcomes in patients after PCI [16, 21, 22].

In the present study, we found that the inflammatory proteins, both post-PCI PTX3 and hsCRP, were significantly associated with long term MACEs in patients with CAD after DES implantation by the univariate Cox regression analysis. However, in the multivariate Cox regression analysis that included PTX3, hsCRP, and other well-known clinical and biochemical predictors which were significant different by univariate Cox regression analysis, PTX3, but not hsCRP, was an independent inflammatory predictor of MACEs. We assessed the association between PTX3 and hsCRP and found that PTX3 was weakly but significantly related with hsCRP (*R* = 0.261, *P* < 0.001) by correlation analysis; this association between them and the higher specificity of PTX3 for localized inflammation and damage in the cardiovascular system [3–5, 7–9] might resulted in the superior prognostic value of PTX3.

Similarly, the serum of PTX3 was weakly correlated with cTnI (*R* = 0.326, *P* < 0.001); this may be PCI induces inflammatory reaction in the injured vessel wall that cause PTX3 increasing [16], and PCI procedure also induces cardiac damage which makes cTnI elevated [23, 24].

Recently, several studies have mentioned that PTX3 was associated with the adverse cardiovascular events in the patients after PCI [17, 18]. Kotooka et al. [17] reported that PCI induced a significant inflammatory reaction in the injured vessel wall and induced the plasma PTX3 concentration increased that lead to the development of neointimal thickening and restenosis in 20 patients after BMS implantation. Hudzik et al. [18] indicated that PTX3 may be a more sensitive marker of local inflammatory response due to vessel injury by BMS than hsCRP and the levels of PTX3 is associated with the MACEs after PCI. DES can greatly reduce incidence of cardiovascular events because of its anti-inflammatory properties compared to BMS [19], whether post-PCI PTX3 is still associated with the cardiovascular events after DES implantation is not known. In the present study, we illustrated that post-PCI PTX3 related with the long-term MACEs and as an independent risk factor in patients after DES implantation.

In addition, our finding that cTnI was associated with MACEs and independence of other risk factors; the finding was consistent with previous studies that cTnI elevation after nonemergent PCI was indicative of an increase in long-term all-cause mortality as well as the composite adverse events of all-cause mortality and MI [23, 24]. Meanwhile, in the present study, we demonstrated that multiple stents was an independent predictor of cardiovascular events which is consistent with prior reports [25, 26].

There were several limitations in this study. First, although this was a prospective study, our analysis was derived from a relative small cohort in a single-center; compared with large clinical trials, this analysis could not be regarded as statistically robust; a large population of patients after DES implantation is necessary to generalize our PTX3 findings. Second, although we assessed the plasma PTX3 levels before and at 24 h after PCI, serial measurements of PTX3 might be more useful for evaluating changes in inflammatory status, estimating risk during the followup period, and directing in- and out-patient treatments. Third, multivariate COX regression modeling although effective at adjusting for variables included in the model cannot adjust for variables not recorded in the database.

## 5. Conclusion

In conclusion, compared with CRP, PTX3 may be a more reliable inflammatory predictor for long-term cardiovascular events in patients after DES implantation. Measurement of plasma PTX3 may substantially improve the early risk stratification of patients undergoing DES implantation.

## Figures and Tables

**Table 1:** Clinical characteristics of patients with and without MACEs.

	All patients (*n* = 596)	Patients without MACEs (*n* = 514)	Patients with MACEs (*n* = 82)	*P* value
Age (years)	65.9 (8.1)	65.3 ± 8.1	69.7 ± 7.3	0.000
Male, *n* (%)	467 (78.4)	402 (78.2)	65 (79.3)	0.886
CCSA class > II, *n* (%)	184 (30.9)	149 (29.0)	35 (42.7)	0.015
LVEF < 50%, *n* (%)	178 (29.8)	145 (28.2)	33 (40.2)	0.037
Diabetes mellitus, *n* (%)	92 (15.4)	73 (14.6)	19 (20.7)	0.047
Hypertension, *n* (%)	319 (53.5)	272 (52.9)	47 (57.3)	0.477
Hyperlipidemia, *n* (%)	192 (32.2)	164 (31.9)	28 (34.1)	0.704
Current smoking, *n* (%)	182 (30.5)	153 (29.8)	29 (35.4)	0.368
cTnI (ng/mL)	0.23 ± 0.16	0.20 ± 0.12	0.37 ± 0.24	0.000
HsCRP (ng/mL)	3.27 ± 1.19	3.14 ± 1.14	4.09 ± 1.16	0.000
In-hospital therapy				
Aspirin, *n* (%)	596 (100)	514 (100)	82 (100)	
Clopidogrel, *n* (%)	596 (100)	514 (100)	82 (100)	
*β*-Blockers, *n* (%)	465 (78.0)	404 (78.6)	61 (74.4)	0.391
ACE-I or ARB, *n* (%)	532 (89.3)	463 (90.1)	69 (84.1)	0.123
Statins, *n* (%)	596 (100)	514 (100)	82 (100)	
Types of DES				
Sirolimus, *n* (%)	390 (65.4)	334 (65.0)	56 (68.3)	0.618
Zotarolimus, *n* (%)	130 (21.8)	113 (22.0)	17 (20.7)	0.886
Paclitaxel, *n* (%)	63 (10.6)	53 (10.3)	10 (12.2)	0.565
Others, *n* (%)	13 (2.2)	10 (1.9)	3 (3.7)	0.403
Multiple stents, *n* (%)	238 (39.9)	190 (37.0)	48 (58.5)	0.0004

CCSA: Canadian Cardiovascular Society Angina; LVEF: left ventricular ejection fraction; PTX3: pentraxin-3; cTnI: cardiac troponin I; HsCRP: high-sensitivity C-reactive protein; ACEI: angiotensin-converting enzyme inhibitor; ARB: angiotensin-receptor blocker.

**Table 2:** Clinical characteristic according to median value of PTX3 at 24 h after PCI.

	PTX3 < 4.384 ng/mL (*n* = 299)	PTX3 > 4.384 ng/mL (*n* = 297)	*P* value
Age (years)	66.27 ± 8.5	65.53 ± 7.8	0.269
Male, *n* (%)	242 (80.9)	225 (75.8)	0.136
CCSA class > II, *n* (%)	86 (28.8)	98 (33.0)	0.288
LVEF < 50%, *n* (%)	76 (25.4)	102 (34.3)	0.020
Diabetes mellitus, *n* (%)	38 (12.7)	54 (18.2)	0.070
Hypertension, *n* (%)	163 (54.5)	156 (52.5)	0.408
Hyperlipidemia, *n* (%)	94 (31.4)	84 (28.3)	0.726
Current smoking, *n* (%)	98 (32.8)	98 (33.0)	0.248
PTX3 before PCI (ng/mL)	3.11 ± 0.62	3.13 ± 0.64	0.699
cTnI (ng/mL)	0.205 ± 0.11	0.251 ± 0.16	0.000
HsCRP (ng/mL)	3.045 ± 1.12	3.496 ± 1.12	0.000
In-hospital therapy			
Aspirin, *n* (%)	299 (100)	297 (100)	
Clopidogrel, *n* (%)	299 (100)	297 (100)	
*β*-Blockers, *n* (%)	238 (79.6)	227 (76.4)	0.374
ACE-I or ARB, *n* (%)	272 (91.0)	260 (87.5)	0.188
Statins, *n* (%)	299 (100)	297 (100)	
Types of DES			
Sirolimus, *n* (%)	197 (65.9)	193 (65.0)	0.863
Zotarolimus, *n* (%)	66 (22.1)	64 (21.5)	0.921
Paclitaxel, *n* (%)	29 (9.7)	34 (11.4)	0.508
Others, *n* (%)	7 (2.3)	6 (2.0)	0.999
Multiple stents, *n* (%)	105 (19.5)	133 (32.9)	0.024

Abbreviations as in Table 1.

**Table 3:** Risk stratification of patients after coronary stent implantation based on increased PTX3 (above the median value of 4.384 ng/mL).

PTX3 (ng/mL)	Overall	>4.384	<4.384	*P*
*n*	596	297	299	
MACEs, *n* (%)	82 (13.8)	61 (20.5)	21 (7.0)	0.000
Cardiac death, *n* (%)	22 (3.7)	14 (4.7)	8 (2.7)	0.200
Nonfatal MI, *n* (%)	14 (2.3)	11 (3.7)	3 (1.0)	0.033
TVR, *n* (%)	46 (7.7)	36 (12.1)	10 (3.3)	0.00008

MACEs: major adverse cardiovascular events; MI: myocardial infarction; TVR: target vessel revascularization.

**Table 4:** Univariate and multivariate Cox regression analysis of major adverse cardiovascular events.

Variables	Univariate analysis RR (95% CI)	*P*	Multivariate analysis RR (95% CI)	P
Age	1.072 (1.040–1.106)	0.000	1.041 (1.008–1.074)	0.013
Male	1.002 (0.587–1.709)	0.994		
CCSA > II	1.762 (1.137–2.730)	0.011	1.009 (0.563–1.808)	0.874
LVEF < 50%	1.662 (1.069–2.585)	0.024	1.061 (0.591–1.906)	0.842
Diabetes mellitus	1.760 (1.053–2.940)	0.031	1.234 (0.677–2.250)	0.493
Hypertension	1.125 (0.725–1.744)	0.599		
Hyperlipidemia	1.096 (0.694–1.731)	0.693		
Current smoking	1.254 (0.797–1.973)	0.327		
PTX3	3.233 (1.968–5.311)	0.000	2.512 (1.466–4.305)	0.001
cTnI	1.029 (1.011–1.047)	0.002	1.012 (1.004–1.284)	0.008
HsCRP	1.832 (1.529–2.195)	0.000	1.093 (0.998–1.197)	0.056
Multiple stents	2.694 (1.724–4.211)	0.000	2.401 (1.479–3.899)	0.0004

Abbreviations as in Table 1.
